# The effect of different pulp-capping materials on proliferation, migration and cytokine secretion of human dental pulp stem cells

**DOI:** 10.22038/ijbms.2020.41511.9814

**Published:** 2020-06

**Authors:** Salma Omidi, Mehdi Bagheri, Mozhgan Fazli, Naghmeh Ahmadiankia

**Affiliations:** 1 Department of Endodontics, Dental School, Mazandaran University of Medical Sciences, Sari, Iran; 2 Clinical Research Development Unit, Imam Hossein Hospital, Shahroud University of Medical Sciences, Shahroud, Iran; 3 School of Medicine, Shahroud University of Medical Sciences, Shahroud, Iran

**Keywords:** Biodentine, Calcium enriched mixture, Human dental pulp stem – cells, Mineral trioxide aggregate, TheraCal

## Abstract

**Objective(s):**

Biocompatibility of dental biomaterials plays a critical role in regeneration of dental stem cells. The aim of present study was to evaluate the effects of novel biomaterials of TheraCal-LC (TheraCal; Bisco), Angelus mineral trioxide aggregate (MTA; Angelus), calcium-enriched mixture (CEM; BioniqueDent), and Biodentine (Septodont) on viability of human dental pulp stem cells (hDPSCs). Moreover, the recruitment of dental pulp stem cells is a prerequisite for regeneration of damaged dentin. Therefore, in this study the effects of mentioned biomaterials on migration of hDPSCs and the secretion of some chemoattractive molecules by these cells were examined.

**Materials and Methods:**

The cell viability of hDPSCs was assessed using MTT assay. Transwell migration assay was used to determine cell migration ability. The cytokine secretion was evaluated using enzyme-linked immunosorbent assay.

**Results:**

The biomaterials of MTA, CEM, and Biodentine at different dilutions had no cytotoxic effects on hDPSCs at different time points; however, non-diluted extract of TheraCal showed toxic effects after 24, 48, and 72 hr. Meanwhile, the highest cell migration was observed in the presence of CEM and Biodentine (*P*<0.05). The secretion of MCP-1 and TGF-β1 were higher in hDPSCs treated with Biodentine compared to some other groups (*P*<0.05, *P*<0.01). Moreover, TheraCal decreased protein secretion of TNF-α (*P*<0.05), and IL-8 (*P*<0.01) in hDPSCs.

**Conclusion:**

The biological compatibility associated with CEM and Biodentine indicates promising applications in the field of vital pulp therapy.

## Introduction

Vital pulp therapy (VPT) is a conservative treatment that involves the removal of local irritants and placement of a protective material directly or indirectly over the pulp. The main objective of this therapy is to maintain the healthy pulp tissue (1). Pulp vitality is extremely important for the tooth viability, since it provides nutrition and acts as biosensor to detect pathogenic stimuli. Moreover, the secondary dentine deposition will continue on preserved vital pulp (2). It is now accepted that a highly proliferative population of progenitor/stem cells resides within dental pulp (3). The repair of pulp-dentin complex after VPT involves the migration and differentiation of dental pulp stem cells (DPSCs) to secondary odontoblasts followed by dentinogenesis and formation of a dentinal bridge sealed on the exposed pulp (4). In recent years, with the progress of regenerative and molecular approaches, it is known that repair of pulp-dentin complex after VPT might be affected by the biological properties of used biomaterials (5-7). Various biomaterials, including TheraCal (8), mineral trioxide aggregate (MTA) (9), calcium-enriched mixture (CEM) (10), and Biodentine (11) have been used as capping agents for VPT.

TheraCal, as a newly developed biomaterial, is a light-cured, resin-modified, calcium silicate-filled base/liner material designed for direct and indirect pulp capping (8, 12). According to the manufacture’s claim, TheraCal formulation consists of tricalcium silicate particles in a hydrophilic monomer that stimulates hydroxyapatite and secondary dentin bridge formation through calcium release and an alkaline pH (13). Moreover, MTA as a calcium silicate-based cement is becoming the material of choice for direct pulp capping in the past decades (9). The MTA mechanism of action is similar to calcium hydroxide, which leads to the formation of calcified bridge. However, MTA has some disadvantages like long setting time, hard handling, discoloration of tooth and high prices (14). Due to these disadvantages, new calcium silicate-based cements such as CEM (10) and Biodentine (11) have been introduced to the market and good clinical outcomes have been reported for them. 

CEM cement is composed of diﬀerent calcium compounds. The particle size of CEM is smaller than MTA, which can justify its good sealing ability (10). Interestingly, CEM induced a thicker dentinal bridge with less pulp inflammation compared to MTA (15). It has also been shown that CEM is biocompatible and induces proliferation and differentiation of human DPSCs (hDPSCs) (4, 16). 

Biodentine is another biomaterial that belongs to the larger group of bioactive materials called bioceramics, which are essentially tricalcium silicate cement. It has a simple application, and its setting time is relatively short compared to the classic portland cement-based materials (17). Recent data imply that Biodentine is a bioactive and biocompatible material capable of enhancing hDPSCs proliferation, migration and adhesion (18). 

The biologic effects of some of these materials were examined individually (4, 13, 14, 19); however, limited studies were performed to evaluate their comparative biological effects on hDPSCs. In this study, we aimed to evaluate and compare the cytotoxic effect of TheraCal, Angelus-MTA, CEM, and Biodentine on hDPSCs. Furthermore, the influence of different material extracts on migration of hDPSCs was examined. 

Moreover, in this study, the release of chemoattractant molecules of monocyte chemoattractant protein-1 (MCP-1), transforming growth factor-β1 (TGF-β1), tumor necrosis factor-α (TNF-α), and interleukin-8 (IL-8) that are likely to play a role in repair of pulp injury after VPT were examined. It was revealed that increased release of MCP-1 by side population (SP) of cells from dental pulp was associated with increased cell migration and angiogenesis leading to higher regenerative potential of dental pulp SP cells (20). Moreover, it was revealed that pulp capping material equipped with TGF-β1 is able to trigger resident stem cells in the pulp to differentiate into odontoblast-like cells and to induce the formation of tertiary dentin (21). Several studies emphasize the role of TNF-α protein in promoting chemotaxis in inflammatory cells and fibroblasts (22, 23). Moreover, the basic biological effect of IL-8 in attracting and activating neutrophils during inflammation was examined (24).

The different secretion of chemoattractant cytokines by hDPSCs under influence of various biomaterials can affect their application *in vivo*.

## Materials and Methods

***Preparation of pulp capping material extracts***

The materials tested in this study were TheraCal LC (Bisco, Lançon De Provence, France), Angelus-MTA (Angelus, Londrina, Brazil), CEM (Bionique Dent, Tehran, Iran), and Biodentine (Septodont, Saint-Maur-des-Fossés). Their complete compositions are described in Table 1. 

The materials were mixed according to the manufacturers’ instructions. Discs of each pulp capping material were shaped under aseptic conditions in 6-well plates (JETBIOFIL, Guangzhou, China) 35 mm in diameter and 2 mm high, sterilized using ultraviolet irradiation for 15 min, and stored in an incubator (Binder GmbH, Tuttlingen, Germany) at 37 ^°^C for 48 hr to achieve complete setting. To prepare material extracts (conditioned medium), the proposed materials were stored in the DMEM (Life Technologies, Carlsbad, CA, USA) for 24 hr at 37 ^°^C in a 95% humidified atmosphere and 5% CO_2_ (5). In accordance with the guidelines of the International Organization for Standardization 10993-5, the ratio of material surface area to medium volume was set at approximately 1.5 cm^2^/ml. The extraction medium was filtered with sterile filters of 0.22 µm pore size (Sartorius A.G. Goettingen, Germany).

***Isolation and culture of hDPSCs***

Freshly extracted teeth were collected from human adult (18 to 25-year-old) patients and placed immediately in sterile Hank’s solution with Pen/Strep. The tooth was obtained with the consent of the patient. Ethics of the study were approved by the ethics committee of Shahroud University of Medical Sciences, Shahroud, Iran. The isolated dental pulps were cut into small pieces and digested in a solution of 3 mg/ml type I collagenase (Sigma) for 3 hr at 37 ^°^C in order to separate cells. Subsequently, the solution was ﬁltered through a 70 mm cell strainer (Becton/Dickinson, Franklin Lakes, NJ, USA). The single cell suspensions were seeded in 35 mm culture dishes and maintained in medium containing DMEM supplemented with 15% fetal bovine serum (Life Technologies) and 1% penicillin- streptomycin (10000 units/ml) (Life Technologies) as antibiotics. Cells were incubated at 37 ^°^C in a 95% humidified atmosphere and 5% CO_2_. The medium was changed every 3 days. Characterization of the DPSCs was performed by ﬂowcytometric analysis and multiple lineage differentiation potential. Primary dental pulp stem cells at passage 3-5 were used in the following experiments.

***MTT assay***

Cytotoxicity of different pulp-capping extracts was evaluated using the 3-(4,5-dimethyl-thiazol)-2,5-diphenyl-tetrazolium bromide (MTT) assay, after 24, 48, and 72 hr of culture. Several dilutions of material extracts (undiluted, 1:2, 1:4, 1:8, 1:16, 1:32) were prepared. Cells were seeded at density equal to 4x10^3^ cells/cm^2 ^in a 96-well plate in 200 µl of different dilutions of material extracts. HDPSCs cultured in DMEM were used as control. At the indicated times, complete culture medium was replaced by serum-free culture medium. MTT (Sigma-Aldrich, St Louis, MO, USA) was added at a final concentration equal to 1 mg/ml and cells were incubated for 4 hr. Then, the culture medium containing MTT was removed and 100 µl dimethyl sulfoxide (DMSO) (Sigma-Aldrich) was added to release formazan. The absorbance at 570 nm was measured using an automatic microplate reader (Stat Fax 2100, Palm City, FL). Each condition was analyzed in quintuplicate. The percentage of the viable cells was calculated using the following equation: (mean OD of treated cells/mean OD of control cells)×100. 

***Migration assay***

Cell migration was assessed using a two-chamber Transwell system with 8 µm pore size and 6.5 mm diameter (SPL. Life Sciences, Pocheon, Korea) as described before (29). Briefly, hDPSCs (1 × 10^4^ cells) suspended in 100 ?l μl of serum free medium were plated on the top chamber of the Transwell, and 500 μl of the material extract prepared with serum-free medium was added to the lower chamber. After incubation at 37 ^°^C in 5% CO_2_ for 24 hr, filters were removed with sterile tweezers and cells that did not migrate through the filter were gently wiped with a cotton swab. Migrating cells beneath the filter were fixed with methanol 100% (Sigma-Aldrich) for 10 min and stained with 0.1 mg/ml DAPI (Sigma-Aldrich). The filters were observed using a fluorescent microscope (IX71 Olympus, Tokyo, Japan). The numbers of the migrating cells in each well were counted in 6 random microscopic fields per filter at 200× magnification. 

***Enzyme-linked immunosorbent assay (ELISA) for MCP-1, TGF-β1, TNF-α, and IL-8***

HDPSCs were seeded in 96-well plates in the conditioned medium for 72 hr. The medium was collected and the levels of secreted MCP-1 (BE51101, IBL, Hamburg, Germany), TGF-β1 (RE51201, IBL), and IL-8 (BE53081, IBL) were determined using immunoassay kits according to the manufacturer’s protocol. The optical density of each well was determined using a microplate reader (Stat Fax 2100, Palm City, FL) at 540 nm. 

***Statistical analysis***

The data were analyzed using one-way ANOVA followed by the Dunnett’s *post hoc* test. Values were compared using multiple comparisons, and *P*-values < 0.05 were considered statistically significant. The values are presented as the mean±standard deviation (SD). GraphPad Prism 6.01 (GraphPad Software, Inc., La Jolla, CA, USA) was used for the statistical analyses. 

## Results

***Cell culture***

**Figure 1:**
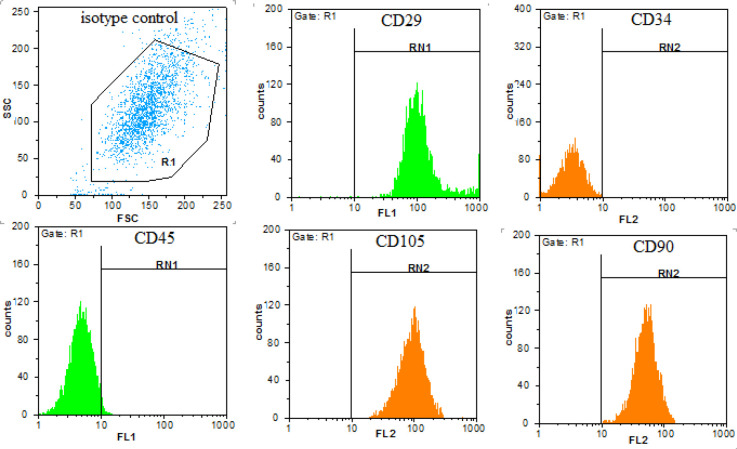
Immunophenotypic characteristics of hDPSCs. Flow cytometric analyses revealed that hDPSCs were positive for cell surface antigens CD29, CD105, CD90, and negative for CD34 and CD45. FITC: ﬂuorescein isothiocyanate, hDPSCs: Human dental pulp stem cells

**Figure 2:**
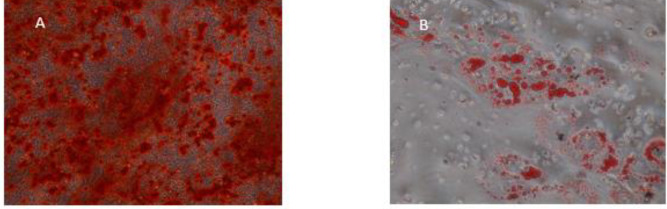
Differentiation capacity of hDPSCs, 20 days after induction with osteogenic and adipogenic medium. (A) Differentiated hDPSCs were stained with alizarin red. (B) hDPSCs induced with adipogenic medium were stained with Oil Red O. These micrographs are representative examples of the experiments. hDPSCs: Human dental pulp stem cells

After isolation and culture of hDPSCs, to conﬁrm the mesenchymal stem cell phenotype of the cells, hDPSCs were characterized by ﬂowcytometry. These cells were CD90+, CD105+, CD 29+, CD34-, and CD45- (Figure 1). Differentiation along adipogenic and osteogenic lineage were performed too (Figure 2). The osteogenic differentiation was confirmed by deposition of alizarin red staining in a mineralized matrix (Figure 2A). Adipogenic differentiation was demonstrated by accumulation of lipid vacuoles in cytoplasm of the cells indicated by Oil Red O staining (Figure 2B). 

***Influence of different material extracts on viability of hDPSCs***

**Figure 3:**
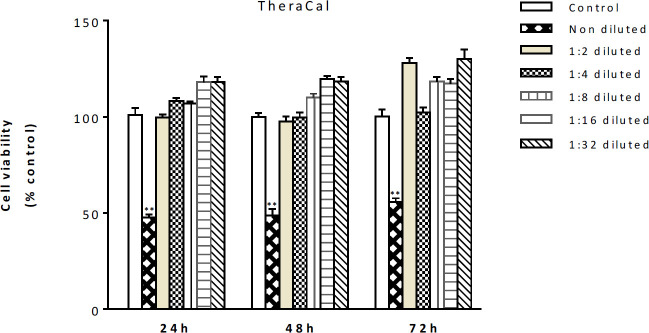
The cytotoxic effects of various dilutions of TheraCal on hDPSCs. Non-diluted extract of TheraCal showed toxic effects on hDPSCs after 24, 48, and 72 hr. ** donates *P*<0.01. hDPSCs: Human dental pulp stem cells

We examined the effects of several dilutions (non-diluted, 1:2, 1:4, 1:8, 1:16, and 1:32) of each biomaterial extract on viability of hDPSCs. The results showed that the biomaterials of MTA, CEM, and Biodentine at different dilutions had no cytotoxic effects on hDPSCs at different time points (data not shown); however, non-diluted extract of TheraCal showed toxic effects after 24, 48, and 72 hr (*P*<0.01) (Figure 3). 

**Figure 4:**
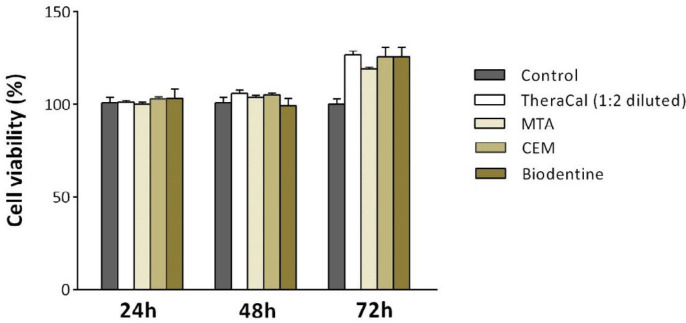
Cell viability as determined using MTT assay. The effects of MTA, CEM, and Biodentine (non-diluted extract) and also TheraCal (1:2 diluted) on cell survival after 24, 48, and 72 hr. MTA: Mineral trioxide aggregate, CEM: Calcium-enriched mixture

Therefore, the following experiments were performed with 1:2 diluted extract of TheraCal to make sure that cytotoxic effect does not interfere with other outcomes. Moreover, the effects of MTA, CEM, and Biodentine (non-diluted extract) and TheraCal (1:2 diluted) on viability of hDPSCs were compared to each other. There was no significant difference in viability of hDPSCs after treatment with these material extracts after 24, 48, and 72 hr (Figure 4). 

***Influence of different material extracts on migration of hDPSCs***

**Figure 5:**
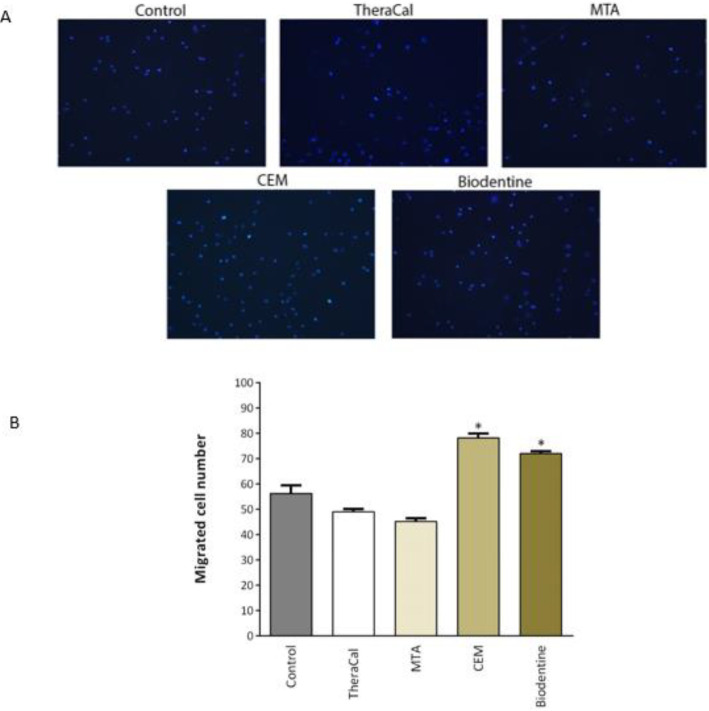
The migration ability of hDPSCs exposed to TheraCal (1:2 diluted), MTA, CEM, and Biodentine extracts as evaluated by transwell migration assay. A) Representative images of migrated cells stained with DAPI. B) The results presented are an average number of migrated hDPSCs on the underside of the filter. * donates *P*<0.05 as compared to the control. MTA: Mineral trioxide aggregate, CEM: Calcium-enriched mixture, hDPSCs: Human dental pulp stem cells

The effects of different material extracts on migration of hDPSCs were evaluated. Interestingly, CEM and Biodentine significantly increased the migration of hDPSCs (*P*<0.05) (Figure 5).

***Influence of different material extracts on secretion of cytokines by hDPSCs***

**Figure 6:**
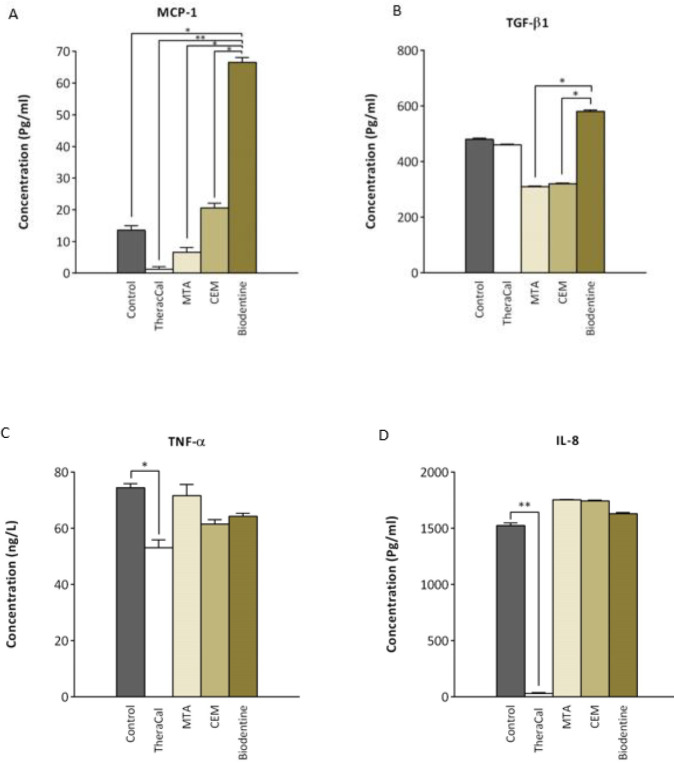
Cytokine release as assessed by ELISA. (A) MCP-1, (B) TGF-β1, (C) TNF-α, and (D) IL-8 concentrations were measured in the supernatant of hDPSCs cultured with TheraCal (1:2 diluted), MTA, CEM, and Biodentine extracts for 72 hr. * and ** donate *P*<0.05 and *P*<0.01, respectively as compared to the control. MCP-1: Monocyte chemoattractant protein-1, TGF-β1: Transforming growth factor-β1, TNF-α: Tumor necrosis factor-α, IL-8: Interleukin-8, MTA: Mineral trioxide aggregate, CEM: Calcium-enriched mixture, hDPSCs: Human dental pulp stem cells

The concentrations of cytokines of MCP-1, TGF-β1, TNF-α, and IL-8 in the supernatant of hDPSCs after 72 hr incubation with the studied material extracts are represented in Figure 6. These results showed that the secretion of MCP-1 is significantly higher in hDPSCs treated with Biodentine compared to other groups (*P*<0.05, *P*<0.01) (Figure 6A). Moreover, our results revealed that the concentration of TGF-β1 in the supernatant of hDPSCs increased after 72 hr in Biodentine group compared to other groups, and this increase is significant between Biodentine with MTA and CEM groups (*P*<0.05) (Figure 6B). 

Additionally, the secretion of TNF-α, and IL-8 by hDPSCs treated with different material extracts were examined. There was no significant difference in the secretion of TNF-α and IL-8 in the supernatant of hDPSCs following 72 hr between the MTA, CEM, and Biodentine groups with the control; however, it was significantly lower in the TheraCal group (*P*<0.05,* P*<0.01) (Figure 6 C,D). 

## Discussion

To promote pulp healing and functional recovery, pulp capping materials should either stimulate cell viability or be biologically neutral (30, 31). Our result shows that there was no significant difference in cell viability of hDPSCs between different groups of MTA, CEM, and Biodentine at different time points. 

Gonzalez *et al.* reported that cell viability in the presence of Biodentine eluates was signiﬁcantly higher than that obtained using MTA Angelus and TheraCal after 48 hr of incubation with stem cells isolated from human exfoliated primary teeth (32). In another study, the effects of MTA, CEM, and Biodentine after different incubation times with human periodontal ligament fibroblasts were evaluated. MTA and CEM presented more than 90% cell viability after 24 and 48 hr of incubation; however, cell viability was decreased significantly after 72 hr of incubation. Biodentine showed significantly less cell viability (73%) after 24 hr of incubation, whereas more than 90% cell viability was observed after 48 and 72 hr of incubation (33). Furthermore, Mozayeni *et al.* reported higher cytotoxicity of CEM cement compared to MTA on L929 fibroblasts at 1, 24 and 168 hr (34). In contrast, some other studies have also shown that CEM cement, similar to MTA, has optimal biocompatibility (35, 36). Using the mouse odontoblast cell line (MDPC-23), Poggio *et al.* reported that Biodentine has better cell biocompatibility than MTA Angelus and ProRoot MTA eluates after 72 hr of culture (37). 

Such controversy in results may be related to the type of studied cells, method of cytotoxicity assessment, direct contact of cells with the materials, concentration of materials and studied time points.

Furthermore, our *in vitro* study showed that undiluted TheraCal extract is toxic to hDPSCs. We assumed that this effect may be attributed to non-polymerization of resinous contents of TheraCal, which can exert toxic effects. This result is in agreement with a previously published work by Jeanneau *et al.*, which showed the toxic effects of TheraCal on human pulp fibroblasts (8). Collado-González *et al.* reported that neither Biodentine nor MTA was cytotoxic for stem cells isolated from human exfoliated primary teeth; however, in the presence of TheraCal LC eluates, cell viability was very low or almost absent (32). Moreover, the clinical efficacy of TheraCal was lower than MTA and Biodentine (38). In contrast, some studies revealed that TheraCal is biocompatible (12) and its cytotoxicity and genotoxicity are comparable to MTA and Biodentine (39). This discrepancy in the results may be due to difference in experimental conditions.

Bakhtiar *et al.* in a clinical trial examination reported that Biodentine and MTA performed better than TheraCal when used as partial pulpotomy agent and presented the best clinical outcomes. They reported more pain, less normal pulp organization and less complete dentin bridge formation in TheraCal group, which may be attributed to non-polymerized monomer in the TheraCal (40).

Further, we examined the effects of TheraCal, MTA, CEM, and Biodentine extracts on migration of hDPSCs. Cellular migration is a critical process for tissue repair in a variety of physiological and pathological conditions (41). It has been suggested that migration and recruitment of hDPSCs happen in the first phase of tooth repair during pulp capping (42). Our study showed that CEM and Biodentine were able to promote the migration of hDPSCs, and this ability was significantly higher compared to TheraCal and MTA materials. Similarly, Tomas-Catala *et al.* showed that Biodentine has a stronger effect on the migration ability of hDPSCs compared to NeoMTA Plus and MTA Repair HP (5). Zhu *et al.* reported increased migration of hDPSCs after treatment with MTA extract; however, we did not observe this effect in our study (7). This different outcome may be related to the type of MTA (which was ProRoot-MTA in their study and Angelus-MTA in ours), different setting time applied to prepare MTA (3 days in their study and 48 hr in ours), and the incubation time to prepare material extract (3 days in their study and 24 hr in ours). These differences could overshadow their biological effects. 

Furthermore, in our study, the secretion of cytokine MCP-1 was examined. MCP-1 belongs to the CC subfamily of chemokines and exerts its effects by binding to CC chemokine receptor 2 (CCR2) (43). In a study by Hayashi *et al*., the role of MCP-1 as a potent trophic factor for dental pulp repair was examined. It was revealed that increased release of MCP-1 by SP of cells from dental pulp was associated with increased cell migration and angiogenesis leading to higher regenerative potential of dental pulp SP cells. Interestingly, they showed that BrdU-positive migrated cells to the site of regeneration express CCR2 (20). MCP-1 has been recognized as an angiogenic factor (44), which may help pulp repair after VPT. Lue *et al.* showed that Biodentine induces increased mRNA expression of MCP-1 in hDPSCs (45). In accordance with previous reports, our results showed that Biodentine increased secretion of MCP-1 by hDPSCs and this amount was significantly higher compared to TheraCal, MTA, and CEM groups. 

Moreover, MCP-1 is one of the key chemokines that regulates migration and infiltration of monocytes/macrophages, which is a typical feature of inflammation (43). Most of the published studies report that the healing sequence starts with an initial moderate inflammatory process, and now there are evidences that inflammation is a prerequisite for tissue healing as a first step, followed by pulp repair (46, 47). We assumed that Biodentine may induce moderate inflammation via induction of hPSCs to secrete inflammatory mediators leading to pulp repair. Nevertheless, this assumption should be confirmed by *in vivo* experiments.

The other investigated cytokine in our study was TGF-β1. Previous data revealed that growth factors such as TGF family released from the dentin matrix can induce the differentiation of progenitor/stem cells into odontoblast-like cells (48). It was confirmed that, MTA effectively increases dentin repair by inducing hDPSCs to secrete TGF-β1 and bone morphogenetic proteins (BMPs) (4). Zhang *et al.* showed that pulp capping material equipped with TGF-β1 is able to trigger resident stem cells in the pulp to differentiate into odontoblast-like cells and to induce the formation of tertiary dentin (21). In this study, we examined the secretion of TGF-β1 by hDPSCs treated with different material extracts, and Biodentine showed the highest level of TGF-β1 release. In accordance with our findings, Laurent *et al* demonstrated that Biodentine induces release of TGF-β1 from human pulp cells (19). Biodentine by inducing TGF-β1 release can enhance dentin formation and improve outcomes of VPT. 

TNF-α is a product of macrophages that populates the inflamed pulp (49). Several studies emphasize the role of TNF-α protein in perpetuating chemotaxis in inflammatory cells and fibroblasts (22, 23). Silva *et al.* reported no significant differences in production of TNF-α amongst MTA, Biodentine and the negative control, which was in accordance with our results (50). In contrast, El Karim *et al.* reported decreased release of TNF-α by odontoblast-like cells following Biodentine treatment (51). This discrepancy may be due to the used method and the type of cells, which were different from our study. IL-8, known as CXCL8 (C-X-C motif chemokine ligand 8), is another chemokine studied in this experiment. The basic biological effect of IL-8 is attracting and activating neutrophils during inflammation (24). Our results revealed that MTA, CEM, and Biodentine do not change the secretion of IL-8 by hDPSCs compared to the control. However, the secretion of TNF-α and IL-8 was significantly decreased in TheraCal group. This may be attributed to the presence of resin in its composition and its lower cytocompatibility (8). It has been reported that up to 50% of methacrylate monomer double bonds remain unreacted in resin polymers (52). The non-polymerized monomers in the composite represent a significant risk when they leach out from the composite and reach dental pulp (53). This indicates that it is impossible to obtain complete polymerization, particularly if the material is applied in a humid environment as in direct pulp capping (38). Similarly, resin components of TheraCal including HEMA, BisGMA, TEGDMA, and UDMA may remain non-polymerized after contact with pulp tissue, and these are known to be not cytocompatible for pulp cells (54). A previous study revealed that TheraCal conditioned medium (0.05 cm^2^/ml) after 24 and 48 hr induced release of IL-8 in cultured fibroblasts and entire tooth cultures (8). This discrepancy in the results may be due to difference in experimental conditions. We used hDPSCs and a much higher concentration of TheraCal and longer incubation time. 

**Table 1:** Compositions of the materials tested in this study

**Material**	**Manufacturer**	**Composition**
TheraCal [25]	Bisco, Lançon De Provence, France	Portland type III cement, poly(ethylene glycol) dimethacrylate, bis-GMA, and barium zirconate
MTA [26]	Angelus, Londrina, Brazil	Powder: tri-calcium silicate, di-calcium silicate, tri-calcium aluminate, ferroaluminate, tri-calcium, calcium oxide, bismuth oxide Liquid: distilled water
CEM [27]	BioniqueDent, Tehran, Iran	Powder: calcium oxide (CaO), sulfur trioxide (SO3), phosphorous pentoxide (P2O5), and silicon dioxide (SiO2) Liquid: distilled water
Biodentine [28]	Saint-Maur-des-Fossés	Powder: tricalcium silicate (Ca3SiO5), dicalcium silicate (Ca2SiO4), calcium carbonate (CaCO3), iron Oxide (Fe2O3), and zirconium oxide (ZrO2). Liquid: water (H2O) with calcium chloride (CaCl2) and soluble polymer (polycarboxylate)

## Conclusion

Our results indicate that the pulp capping materials including Angelus MTA, CEM, and Biodentine exhibited less cytotoxic effects on hDPSCs than TheraCal. It seems that CEM and Biodentine are promising alternative materials in clinical situations where accelerated hDPSCs migration is required for dentin repair. Nevertheless, future *in vitro* and *in vivo* studies should be performed to add more information on the behavior of new endodentic materials such as Angelus MTA, CEM, Biodentine, and TheraCAl.
